# Stable Carbon Isotope Fractionation during Bacterial Acetylene Fermentation: Potential for Life Detection in Hydrocarbon-Rich Volatiles of Icy Planet(oid)s

**DOI:** 10.1089/ast.2015.1355

**Published:** 2015-11-01

**Authors:** Laurence G. Miller, Shaun M. Baesman, Ronald S. Oremland

**Affiliations:** U.S. Geological Survey, Menlo Park, California.

## Abstract

We report the first study of stable carbon isotope fractionation during microbial fermentation of acetylene (C_2_H_2_) in sediments, sediment enrichments, and bacterial cultures. Kinetic isotope effects (KIEs) averaged 3.7 ± 0.5‰ for slurries prepared with sediment collected at an intertidal mudflat in San Francisco Bay and 2.7 ± 0.2‰ for a pure culture of *Pelobacter* sp. isolated from these sediments. A similar KIE of 1.8 ± 0.7‰ was obtained for methanogenic enrichments derived from sediment collected at freshwater Searsville Lake, California. However, C_2_H_2_ uptake by a highly enriched mixed culture (strain SV7) obtained from Searsville Lake sediments resulted in a larger KIE of 9.0 ± 0.7‰. These are modest KIEs when compared with fractionation observed during oxidation of C_1_ compounds such as methane and methyl halides but are comparable to results obtained with other C_2_ compounds. These observations may be useful in distinguishing biologically active processes operating at distant locales in the Solar System where C_2_H_2_ is present. These locales include the surface of Saturn's largest moon Titan and the vaporous water- and hydrocarbon-rich jets emanating from Enceladus. Key Words: Acetylene—Fermentation—Isotope fractionation—Enceladus—Life detection. Astrobiology 15, 977–986.

## 1. Introduction

Acetylene (C_2_H_2_) is formed in planetary atmospheres primarily by photochemical conversion of methane (Kasting *et al.*, 1983; Zahnle, 1986). Methane (CH_4_) and hence acetylene were abundant in the anoxic atmosphere of early Earth (Kasting, 2005). Both gases have been identified in comets (Brooke *et al.*, 1996; Mumma *et al.*, 2005), thereby adding organic material to early Earth (Chyba *et al.*, 1990) and throughout the Solar System. Today, terrestrial tropospheric concentrations of C_2_H_2_ are generally less than 0.1 ppbv, a level which is mostly supported by anthropogenic leakage. Minor amounts of C_2_H_2_ are produced during biomass burning (Andrea and Merlet, 2001) and by methanogenic attack of halogenated hydrocarbons (Belay and Daniels, 1987). Much higher C_2_H_2_ concentrations (ppmv and higher levels) are found within the atmospheres of a number of jovian-type planets and satellites in our solar system (Macy, 1980; Noll *et al.*, 1986; Waite *et al.*, 2007; Oremland and Voytek, 2008). For example, Titan's upper atmosphere contains abundant C_2_H_2_ (Shemansky *et al.*, 2005), the condensation and sedimentation of which (Schulze-Makuch and Grinspoon, 2005) leads to concentrations up to 1% in the liquid hydrocarbon lakes on its surface (Cordier *et al.*, 2009). The vaporous plumes emanating from the south polar region of Enceladus, Saturn's sixth largest moon, may contain C_2_H_2_ along with other organic compounds, water, and salts (Waite *et al.*, 2006; Postberg *et al.*, 2011). Matson *et al.* (2007) proposed that C_2_H_2_ originates within Enceladus by thermal processes, with a liquid water body below the icy surface in contact with a hot silicate core. External sources of C_2_H_2_ have also been suggested (Waite *et al.*, 2009; McKay *et al.*, 2012).

There are few chemical reactions that consume C_2_H_2_. Acetylene is used commercially as a feedstock for the production of other organic compounds via catalytic cyclization (Reppe *et al.*, 1969). Removal of traces of C_2_H_2_ from the product stream is performed industrially at elevated temperatures using palladium catalysts often containing other metals (Studt *et al.*, 2008). A more familiar process, combustion, requires a strong oxidant such as O_2_ or N_2_O and an ignition source. Pyrolysis of C_2_H_2_ has also been shown to occur in anoxic aqueous solutions under intense ultrasound irradiation (Hart *et al.*, 1990). Note that the above chemical reactions all require elevated concentrations of C_2_H_2_ or large expenditures of energy in order to proceed. Reaction with hydroxyl radical has been demonstrated (Breen and Glass, 1971); however, this process is unlikely to be important in our troposphere where C_2_H_2_ mixing ratios are low.

Biological processes consume C_2_H_2_ over a range of concentrations and environmental conditions. Aerobic oxidation of C_2_H_2_ by the bacterium *Nocardia rhodochrous* (now called *Rhodococcus rhodochrous*) was documented by Kanner and Bartha (1979) followed by the observation of anaerobic C_2_H_2_ fermentation when using cell-free extracts of *Rhodococcus* A1 (de Bont and Peck, 1980). Anaerobic oxidation of C_2_H_2_ in enrichment cultures derived from estuarine sediments (Culbertson *et al.*, 1981) resulted in the production of CO_2_, and a hypothesized role of C_2_H_2_ as a potential source of carbon and energy for primordial and extraterrestrial food chains was suggested (Culbertson *et al.*, 1988; Oremland and Voytek, 2008). McKay and Smith (2005) calculated that hydrogenation of C_2_H_2_ to CH_4_ could support alien methanogenic life on the surface of Titan at 90 K.

Fermentation of C_2_H_2_ has been well studied by using the model organism *Pelobacter acetylenicus* (Schink, 1985) from which the acetylene hydratase enzyme (AH) was isolated and purified (Rosner and Schink, 1995; Meckenstock *et al.*, 1999; Seiffert *et al.*, 2007). This enzyme is uniquely specific for acetylene and will not react with other compounds (tenBrink *et al.*, 2011). No cross-reactivity was found between antibodies raised against *P. acetylenicus* and cell-free extracts of aerobic acetylene consumers (Rosner *et al.*, 1997), suggesting that aerobic and anaerobic forms of AH are structurally different.

Miller *et al.* (2013) conducted a survey of AH activity in likely anoxic environments and in addition developed a genetic method of AH detection based on gene amplification from extracted DNA using a degenerate primer derived from *P. acetylenicus.* Few (21%) of the environments tested showed AH activity, while fewer still (10%) tested positive for the AH gene. They suggested insufficient degeneracy in their primer design as an explanation for this observation but offered the possibility that other metabolic pathways could be operative in those cases where AH activity was present without detection of the AH gene. They highlighted two cultures as examples of (1) organisms that fermented C_2_H_2_ and contained the AH gene (SFB93, from San Francisco Bay) and (2) organisms that fermented C_2_H_2_ but lacked the AH gene (SV7, from Searsville Lake, CA). These two cultures, sediments, and sediment enrichments from the two environments are the subject of further examination presented in this study.

Patterns of stable carbon isotope fractionation have long been used to distinguish biological processes from purely physical/chemical processes involving carbon compounds. Examples include numerous studies on the provenance of natural hydrocarbons in Earth's crust and sediments (Rosenfeld and Silverman, 1959; Schoell, 1980, 1988; Pohlman *et al.*, 2009). The two principle biochemical pathways of CH_4_ formation (fermentation and CO_2_ reduction) result in different biological fractionation factors (α = κ_12_/κ_13_), and the gas produced can be further distinguished from thermogenic methane by using stable isotopes alone (Whiticar *et al.*, 1986). Consumption of CH_4_ results in smaller kinetic isotope effects (KIE = ɛ or [α − 1] × 1000) for abiotic reaction with OH radicals in the troposphere (Rust and Stephens, 1980; Cantrell *et al.*, 1990) than for microbial oxidation in oxic or anoxic aquatic environments (Whiticar, 1999; Templeton *et al.*, 2006; Kinnaman *et al.*, 2007). Similarly, the carbon KIE for chemical uptake of methyl bromide was significantly lower than the KIE for microbial oxidation in pure cultures, enrichment cultures, and in agricultural field fumigation experiments and soil microcosms (Miller *et al.*, 2001, 2004; Bill *et al.*, 2002). Conversely, larger KIEs were observed for abiotic transformation of the chlorinated ethene TCE (Bill *et al.*, 2001; Slater *et al.*, 2002) than were achieved during microbial reductive dehalogenation of this compound (Bloom *et al.*, 2000; Liang *et al.*, 2007). In addition, abiogenic hydrocarbon production by various mechanisms may result in isotopically depleted products (Sherwood Lollar *et al.*, 2008) giving the appearance of biogenic fractionation. Thus, disparate stable isotope fractionation patterns must be evaluated in concert with additional information to distinguish abiotic from biogenic pathways of production or consumption.

In addition to their use in inferring the pathway of degradation, stable carbon isotopes are used to determine the degree of organic contaminant transformation during degradation by observing the relative enrichment of ^13^C in reactive substrates (Meckenstock *et al.*, 2004; Elsner *et al.*, 2005; Hofstetter *et al.*, 2008). Using this approach, the amount of chlorinated ethene biodegradation via various pathways may be distinguished by using stable isotope fractionation patterns coupled with reactive transport modeling. The assessment of biological and chemical KIEs is an important first step in applying patterns of stable carbon isotope fractionation to distinguish biological from nonbiological processes or to discern the degree of transformation of carbon compounds. In the case of C_2_H_2_, there are few important chemical reactions to consider; hence, we investigated biological KIEs. This study reports the first documented stable carbon isotope fractionation of C_2_H_2_ during fermentation by estuarine sediments, sediment enrichments derived from freshwater mud, and pure cultures obtained from estuarine sediments.

## 2. Materials and Methods

### 2.1. Sediments and enrichments

Estuarine sediments were collected at low tide from the surface of an intertidal mudflat in San Francisco Bay (Culbertson *et al.*, 1981; Miller *et al.*, 2013). Sediment slurries were prepared under flowing N_2_ by mixing sediment with artificial mineral salts media (ABW; Culbertson *et al.*, 1981) in a large beaker in a ratio of 1 part sediment to 5 parts ABW. Freshwater lake sediments were collected by Ekman Grab from Searsville Lake, Stanford, California, at a water depth of 5 m. Sediment slurries were prepared similarly using freshwater media (SeFr1 and SeFr2; Miller *et al.*, 2013).

Acetylene fermenters were enriched from incubated sediment slurries of San Francisco Bay and Searsville Lake that demonstrated C_2_H_2_ consumption (Table 1). A complete description of the enrichment and cultivation of strains SFB93 and SV7 was presented by Miller *et al.* (2013). Amplification and sequencing of the 16S rRNA gene revealed SFB93 to be composed of a single clone aligning within the pelobacter clade and having 96% sequence similarity to *Pelobacter acetylenicus* (GenBank accession number JQ085863). By contrast, SV7 defied purification into a single isolate. At the time of publication (Miller *et al.*, 2013), SV7 consisted of several (perhaps six) species of bacteria, including actinobacteria and a sulfurospirillum-like organism, but no pelobacters.

**Table 1.** Sediments, Sediment Enrichments, and Cultures Derived from Sediments Representing Various Stages of Purification

*Sample*	*Date collected*	*Location*	*Steps taken toward purification*	*Number of organisms*
SFB5	12/19/07	San Francisco Bay Palo Alto, CA N 37°27′28.13″ W 122°06′03.73″	None; this is the original slurry in ABW^a^.	Not tested
SFB93	12/19/07	San Francisco Bay Palo Alto, CA N 37°27′28.13″ W 122°06′03.73″	9 transfers from original slurry in ABW + 1.6 m*M* cysteine-HCl. Transfer to agar plate. Pick individual colony from agar and grow on ABW + vitamins and trace elements.	1
SVM	2/22/12	Searsville Lake Stanford, CA N 37°24′26.63″ W 122°14′16.98″	4 transfers from original slurry in SeFr2^b^.	Not tested
SV7	6/24/08	Searsville Lake Stanford, CA N 37°24′26.63″ W 122°14′16.98″	9 transfers from original slurry in SeFr1^c^ + 1 m*M* sulfide. Final transfer to SeFr2^b^ + 1 m*M* sulfide. Transfer to agar plate. Pick individual colony from agar and perform dilution to extinction in SeFr2. Choose e^5^ tube and grow on SeFr2 + vitamins and trace elements.	≥6

^a^ ABW = Artificial Bay Water media.

^b^ SeFr2 = Fresh Water media lacking ${\rm SO}_4^{\,\,\,2 -}$.

^c^ SeFr1 = Fresh Water media with 2.2 m*M* ${\rm SO}_4^{\,\,2 -}$.

Acetylene used throughout this study was generated by the reaction of calcium carbide with water:

\begin{align*}{\rm CaC}_2 + 2{\rm H}_2{\rm O} \rightarrow {\rm C}_2{\rm H}_2 + {\rm Ca}( {\rm OH})_2 \tag{1}\end{align*}

Two liters of C_2_H_2_ were purified by cryogenic transfer using liquid nitrogen through water vapor traps at −87°C in a vacuum line, followed by pumping of noncondensable gas. The purified gas was frozen into several serum bottles for use in subsequent experiments. Acetylene introduced into the headspace of tubes or serum bottles was distributed between liquid and headspace volumes according to Henry's law such that about two-thirds of the gas was dissolved and one-third remained in the headspace after 20 min equilibration. Incubations began with initial headspace concentrations of 90–240 μmol L^−1^ C_2_H_2_.

### 2.2. Incubations

San Francisco Bay sediment slurries (SFB5; 20 mL) were dispensed under flowing N_2_ to serum bottles (160 mL total volume) containing 10 mL ABW and stoppered before flushing with oxygen-free N_2_ for an additional 5 min. A single heat-killed control was prepared by autoclaving (121°C, 203 kPa for 1 h). Incubations were started by addition of 0.5 mL C_2_H_2_ to the headspace and were allowed to equilibrate for 20 min before initial headspace samples were collected. Slurries were incubated in the dark at 28°C with rotary shaking at 120 rpm. Subsequent headspace samples were collected approximately hourly for 7 h.

Cultures of acetylene fermenters were grown on mineral salts media with added SL10 trace elements (Widdel *et al.*, 1983) and vitamins (Oremland *et al.*, 1994) with cysteine-HCl (SFB93) or Na_2_S (SV7) as reducing agent (Table 1). Strain SFB93 was harvested in late exponential phase for incubation either in growth mode or as a washed-cell suspension. Manipulation of cultures took place in an anaerobic chamber (Type A, Coy Laboratory Products, Ann Arbor, MI) under a mixture of 5% H_2_, 5% CO_2_, and 90% N_2_. For growth experiments, SFB93 (10 mL) was transferred directly to Balch tubes (37 mL), stoppered, and flushed with oxygen-free N_2_ for 5 min. Cell densities were determined by direct cell counting using acridine orange epifluorescence microscopy (Hobbie *et al.*, 1977). Initial cell density was 3.0 × 10^7^ cells cm^−3^. Final cell density was not determined. For washed-cell experiments, cells were centrifuged (7000*g*) and washed twice and then resuspended in a mineral salts medium that lacked trace metals and vitamins before transferring 30 mL to 160 mL serum bottles and flushing with N_2_. Initial cell density was 1.3 × 10^8^ cells cm^−3^. Final cell density was not determined. Triplicate experimental tubes and a single heat-killed control were prepared for each condition. Incubations were started by addition of 0.2 mL C_2_H_2_ to the headspace of tubes and 0.5–1.0 mL C_2_H_2_ to the headspace of serum bottles. Incubations were conducted in the dark at 28°C and 14°C with rotary shaking at 120 rpm.

Sediment enrichments were evaluated at various points along the pathway to purification. Searsville Lake sediment collected from the same site as SV7 but on a different sampling date was transferred four times from slurry using SeFr2 media. This enrichment (SVM) produced up to 7 mmol L^−1^ methane during C_2_H_2_ consumption. The mixed culture was grown to late exponential phase, and 10 mL was transferred to three serum bottles (160 mL) containing 20 mL SeFr2 for incubation at 28°C as above with addition of 0.5 mL C_2_H_2_ to start.

Enrichment strain SV7 was harvested during exponential phase for incubation in growth mode. A 10 mL aliquot of the growing culture was transferred in the anaerobic chamber into 160 mL serum bottles containing 20 mL medium (Rosner *et al.*, 1997). Bottles were stoppered and flushed with oxygen-free N_2_ prior to the start of incubations. Initial cell densities ranged from 1.8 × 10^7^ to 4.8 × 10^7^ cells cm^−3^. Incubations were started by addition of 0.5 mL C_2_H_2_ to the headspace of bottles. Six replicate experimental bottles and one heat-killed control were incubated in the dark with shaking at 28°C.

### 2.3. Analytical

Measurements of headspace concentration and stable carbon isotopic composition were made separately and simultaneously at each time point to determine the KIE due to acetylene fermentation. Headspace C_2_H_2_ concentrations were quantified by flame ionization gas chromatography (Miller *et al.*, 1997, 2013). Precision of FID analyses was ±3% of the reported concentration. The concentration of C_2_H_2_ in each bottle was determined by comparison with standards prepared by dilution of 100% purified C_2_H_2_.

The stable carbon isotopic composition (δ^13^C vs. VPDB) was determined by isotope-ratio-monitoring gas chromatography–combustion–mass spectrometry (GC-C-IRMS) using a HP 5890 gas chromatograph (Agilent Technologies, Santa Clara, CA) fitted with a GS-CARBONPLOT capillary column (30 m × 0.32 mm × 3.0 μm film thickness; J&W Scientific, Agilent Technologies, Santa Clara, CA) connected to an Elementar IsoPrime mass spectrometer through a CuO/NiO combustion interface and a Nafion (Perma Pure, Toms River, NJ) water trap (Kalin *et al.*, 2001; Bill *et al.*, 2002). The CO_2_ monitoring gas measured with each analysis was calibrated against a range of international standards, including NBS-19 and NBS-22 (1.95‰ and −30.0‰ vs. VPDB, respectively).

Precision of the measured δ^13^C values of acetylene was ±0.5‰. Accuracy was evaluated by independently measuring the acetylene reference gas with elemental analyzer–isotope ratio mass spectrometry (EA-IRMS), the spectrometer configured such that a Carlo Erba NA 1500 elemental analyzer was connected to an Elementar Optima mass spectrometer. The δ^13^C values of C_2_H_2_ were corrected to an EDTA working standard calibrated against a range of international standards, including NBS-19 and NBS-23 (−35.5‰ vs. VPDB). The δ^13^C values for the acetylene reference gas analyzed by GC-C-IRMS were consistently depleted in ^13^C (1.7 ± 0.6‰) compared with the EA-IRMS values. No correction for this discrepancy was made.

Kinetic isotope effects were calculated from the slope of the regression of the logarithm of the fraction of reactant acetylene remaining [-ln(C/C_0_)] against the δ^13^C value at the corresponding time point. Concentration and isotopic composition data from at least three replicates for each live condition were used to determine the slope defining the KIE for each bottle or tube. The errors reported in the text are the standard deviation of the replicate slopes.

## 3. Results

### 3.1. SFB5 slurries

**FIG. 1.**
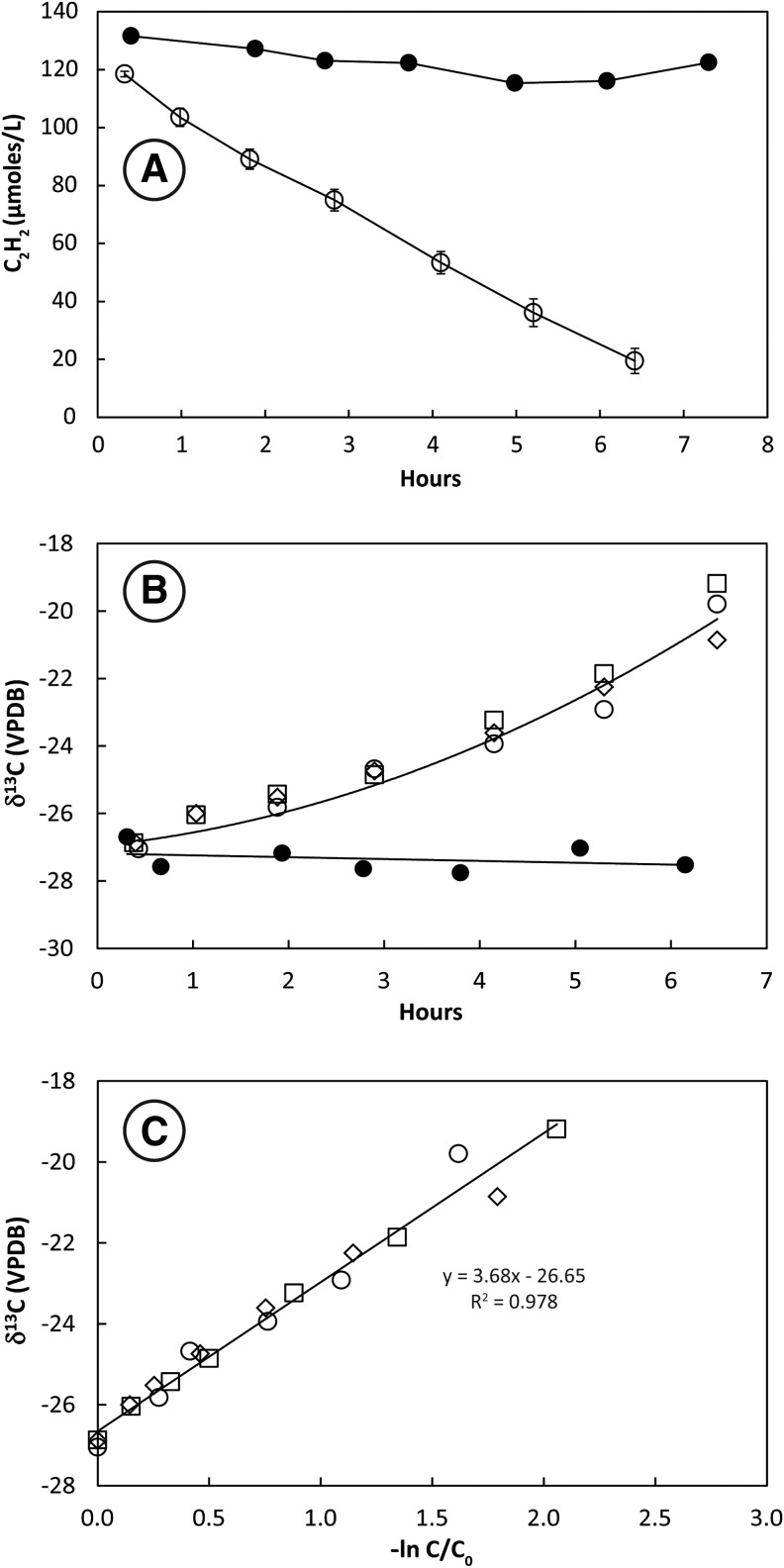
Uptake of C_2_H_2_ (**A**), change in δ^13^C_2_H_2_ with time (**B**), and KIE plot (**C**) for San Francisco Bay sediment slurries SFB5 incubated at 28°C. Results of a single heat-killed control are shown as closed symbols in (A) and (B). Open symbols in (A) represent the mean of triplicate live bottles. Error bars represent ±1 standard deviation. Open symbols in (B) and (C) represent measurements of individual bottles. A linear regression of all analyses along with the slope and *R*^2^ of the fit is shown in (C) for KIEs measured at 28°C.

Headspace C_2_H_2_ concentrations decreased monotonically over ∼7 h in bottles containing San Francisco Bay sediment slurries (Fig. 1A). C_2_H_2_ remained constant in the autoclaved control. Stable carbon isotopes of C_2_H_2_ (δ^13^C_2_H_2_) increased from initial values around −27‰ in three live bottles but remained constant in the autoclaved control (Fig. 1B). The slope of the plotted line of δ^13^C_2_H_2_ against −ln(C/C_0_) for three live bottles (Fig. 1C) shows that the KIE for incubations at 28°C ranged from ɛ = 3.3 to 4.2 and averaged 3.7 ± 0.5‰.

### 3.2. SFB93 under growth conditions

**FIG. 2.**
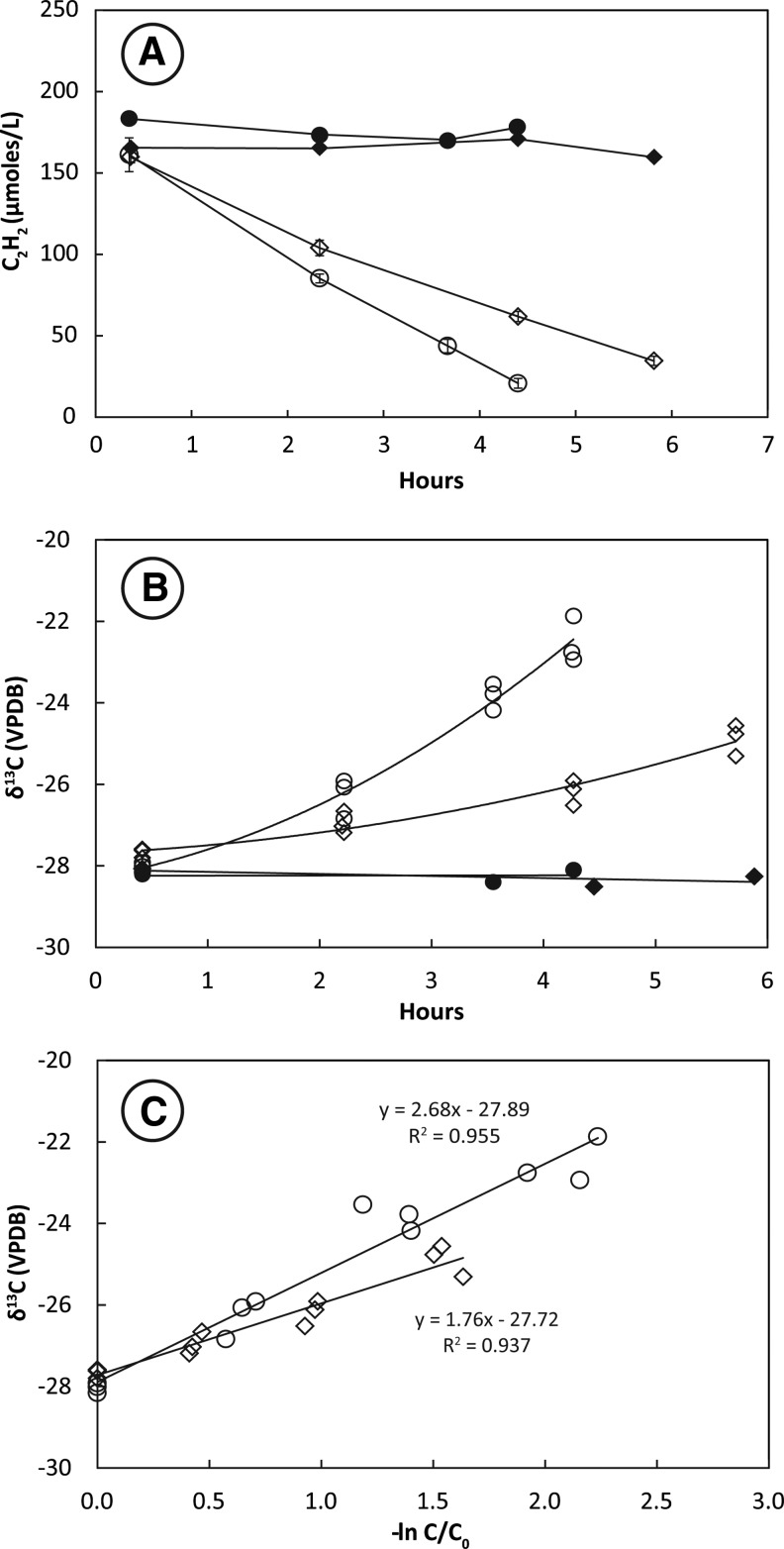
Uptake of C_2_H_2_ (**A**), change in δ^13^C_2_H_2_ with time (**B**), and KIE plot (**C**) for pure-culture SFB93 incubated under growth conditions. Results of single heat-killed control are shown as closed symbols in (A) and (B). Open symbols in (A) represent the mean of triplicate live tubes incubated at 28°C (circles) and 14°C (diamonds). Error bars represent ±1 standard deviation. Open symbols in (B) and (C) represent measurements of individual tubes. A linear regression of all analyses along with the slope and *R*^2^ of the fit is shown in (C) for KIEs measured at 28°C (circles) and 14°C (diamonds).

Removal of C_2_H_2_ from the headspace of tubes containing live cultures was nearly linear over the 5–6 h of uptake (Fig. 2A). Cultures incubated at 28°C consumed C_2_H_2_ more rapidly than those incubated at 14°C. Headspace C_2_H_2_ concentrations remained constant in the autoclaved controls. Stable carbon isotopes of C_2_H_2_ (δ^13^C_2_H_2_) increased from initial values around −28‰ in tubes containing live cultures (Fig. 2B). This increase was greater in cultures incubated at 28°C than in cultures incubated at 14°C. There was no change in the isotopic composition of C_2_H_2_ in heat-killed controls. The slope of the plotted line of δ^13^C_2_H_2_ against −ln(C/C_0_) for live tubes (Fig. 2C) shows that the KIE for incubations at 28°C (ɛ = 2.7 ± 0.3‰) was greater than the KIE for incubations at 14°C (ɛ = 1.8 ± 0.3‰).

### 3.3. SFB93 under washed cell conditions

**FIG. 3.**
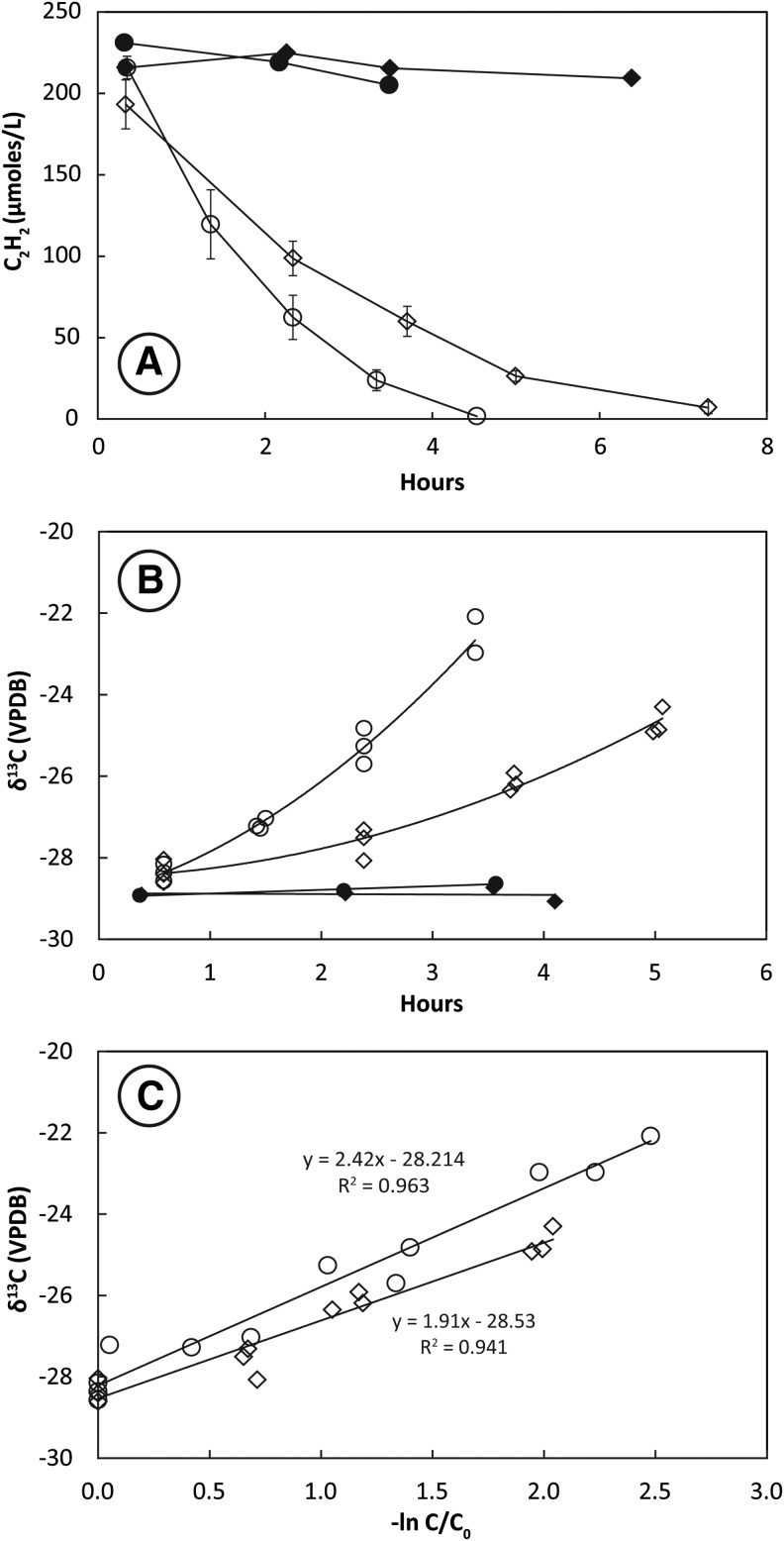
Uptake of C_2_H_2_ (**A**), change in δ^13^C_2_H_2_ with time (**B**), and KIE plot (**C**) for pure-culture SFB93 incubated under washed cell conditions. Results of heat-killed controls are shown as closed symbols in (A) and (B). Open symbols in (A) represent the mean of triplicate live bottles incubated at 28°C (circles) and 14°C (diamonds). Error bars represent ±1 standard deviation. Open symbols in (B) and (C) represent measurements of individual bottles. A linear regression of all analyses along with the slope and *R*^2^ of the fit is shown in (C) for KIEs measured at 28°C (circles) and 14°C (diamonds).

Headspace C_2_H_2_ concentrations decreased rapidly in bottles containing live washed cultures resuspended at high cell density in mineral salts media (Fig. 3A). Cultures incubated at 28°C consumed C_2_H_2_ more rapidly than cultures incubated at 14°C. Headspace C_2_H_2_ concentrations remained constant in the autoclaved controls. Values of δ^13^C_2_H_2_ increased faster in cultures incubated at 28°C than in cultures incubated at 14°C (Fig. 3B). There was no change in the isotopic composition of C_2_H_2_ in heat-killed controls. The slope of the plotted line of δ^13^C_2_H_2_ against −ln(C/C_0_) for live bottles (Fig. 3C) shows that the KIE for incubations at 28°C (ɛ = 2.6 ± 0.2‰) was greater than the KIE for incubations at 14°C (ɛ = 1.9 ± 0.3‰).

### 3.4. Searsville Lake methanogenic enrichments (SVM)

**FIG. 4.**
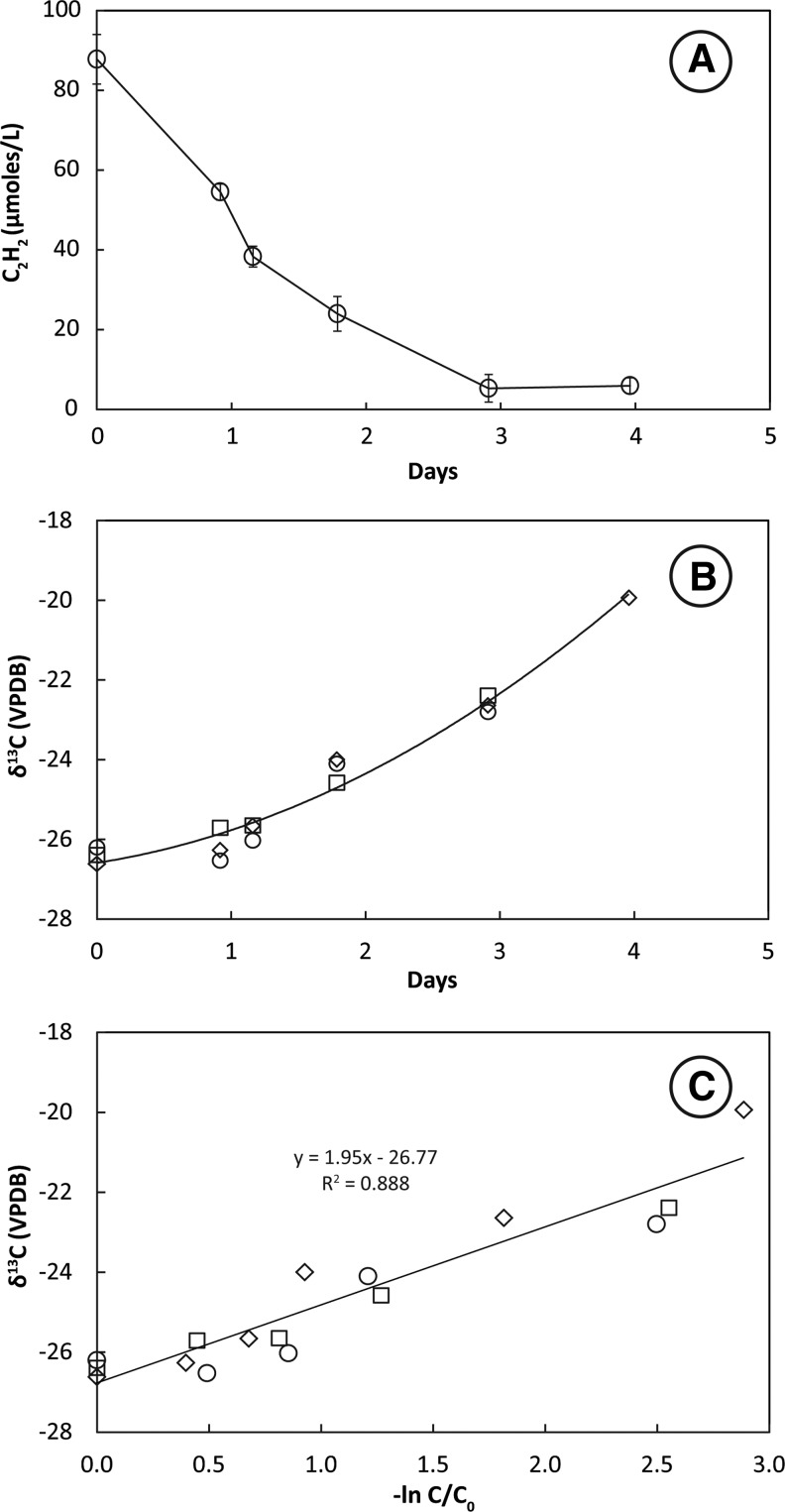
Uptake of C_2_H_2_ (**A**), change in δ^13^C_2_H_2_ with time (**B**), and KIE plot (**C**) for methanogenic mixed culture SVM incubated under growth conditions. Open circles in (A) represent the mean of triplicate live bottles incubated at 28°C. Error bars represent ±1 standard deviation. Open symbols in (B) and (C) represent measurements of individual bottles. A linear regression of all analyses along with the slope and *R*^2^ of the fit is shown in (C) for KIEs measured at 28°C.

Headspace C_2_H_2_ concentrations decreased slowly in bottles containing mixed culture enrichments derived from sediment transfers. A loss of 90 μmol L^−1^ C_2_H_2_ from the headspace over 3–4 days was accompanied by an increase of 20 μmol L^−1^ CH_4_ (data not shown). Values of δ^13^C_2_H_2_ increased slowly in mixed culture enrichments (Fig. 4B). The slope of the plotted line of δ^13^C_2_H_2_ against −ln(C/C_0_) for three live bottles (Fig. 4C) shows that the KIE for incubations at 28°C ranged from ɛ = 1.6 to 2.4 and averaged 1.9 ± 0.5‰.

### 3.5. SV7 under growth conditions

**FIG. 5.**
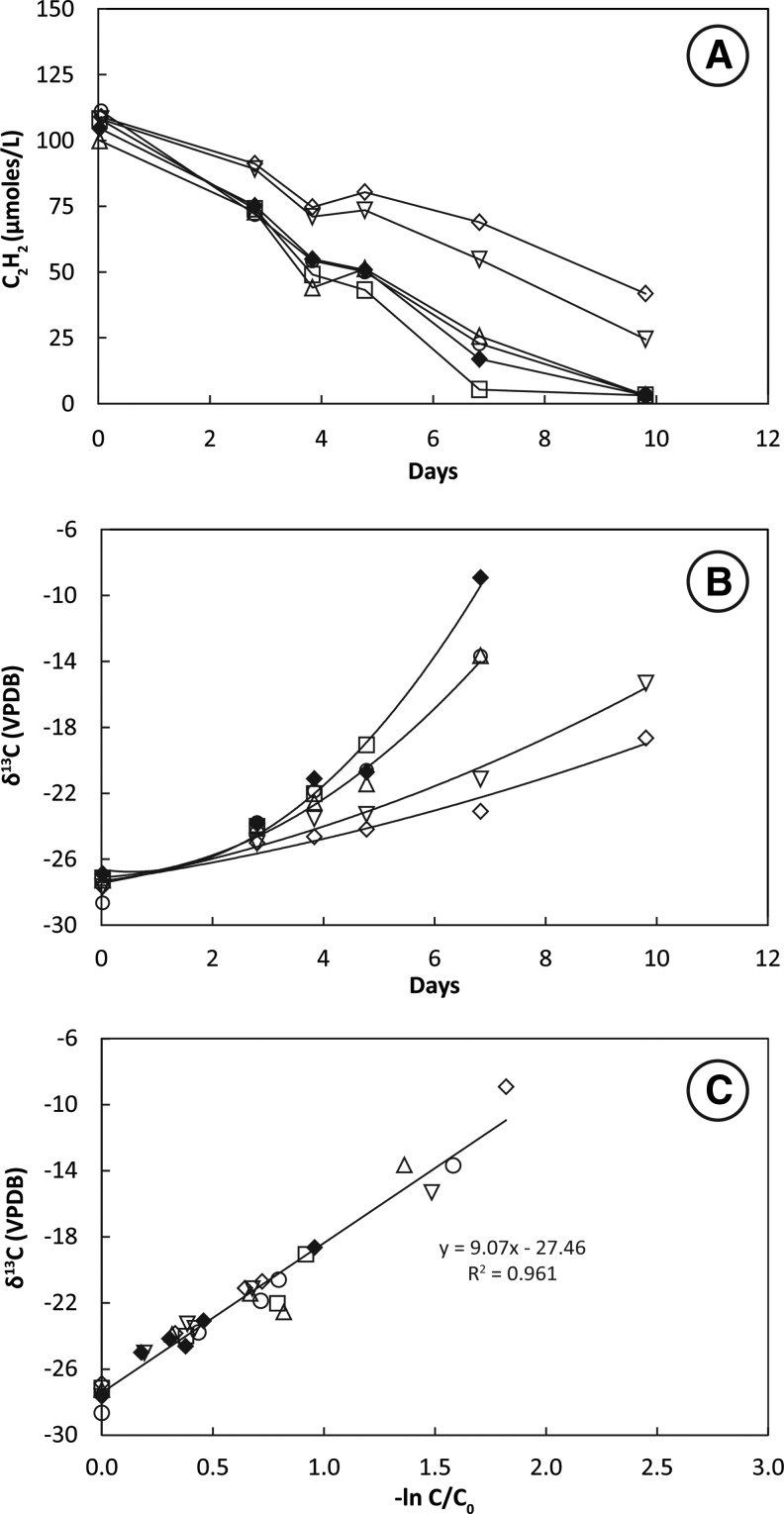
Uptake of C_2_H_2_ (**A**), change in δ^13^C_2_H_2_ with time (**B**), and KIE plot (**C**) for mixed culture SV7 incubated under growth conditions. Symbols represent six individual live bottles incubated at 28°C. A linear regression of all analyses along with the slope and *R*^2^ of the fit is shown in (C) for KIEs measured at 28°C.

Headspace C_2_H_2_ concentrations decreased very slowly in bottles containing highly enriched mixed culture SV7. Removal of 100 μmol L^−1^ C_2_H_2_ from the headspace of live bottles occurred over 7–10 days (Fig. 5A). During this time, δ^13^C_2_H_2_ increased from an initial value of −28‰ to values as high as −9‰ (Fig. 5B). The slope of the plotted line of δ^13^C_2_H_2_ against −ln(C/C_0_) for six live bottles shows the KIE for incubations at 28°C ranged from ɛ = 7.9 to 9.9 and averaged 8.9 ± 0.8‰ (Fig. 5C).

## 4. Discussion

Persistent and uniform KIEs for C_2_H_2_ fermentation were obtained during incubations of San Francisco Bay sediment, enrichments from Searsville Lake sediment, and pure cultures of SFB93 (*Pelobacter* sp.). KIEs measured by primary substrate loss (in this case, C_2_H_2_ loss) reflect all downstream reactions, but KIEs are most influenced by those steps that are rate limiting. Acetylene fermenters such as *P. acetylenicus* carry out sequential exothermic reactions during growth on C_2_H_2_, consisting first of the AH reaction followed by one involving acetaldehyde dismutase (Schink, 1985):

\begin{align*}{\rm C}_2{\rm H}_2 + {\rm H}_2{\rm O} \rightarrow {\rm CH}_3{\rm CHO} \tag{2}\end{align*}

\begin{align*}{\rm CH}_3{\rm CHO} + {\rm H}_2{\rm O} \rightarrow {\rm CH}_3{\rm CH}_2{\rm OH} + {\rm CH}_3{\rm COO}^{-} + {\rm H}^{+} \tag{3}\end{align*}

Additional downstream reactions in sediments and sediment enrichments may utilize the ethanol or acetate formed by Reaction 3 above. These reactions include terminal electron accepting processes such as sulfate reduction and methanogenesis. It is not known which of the above reactions is primarily responsible for the fractionation of carbon isotopes observed. In any case, the fractionation we measured (KIE∼3‰) is neither as large as the KIEs determined for anaerobic or aerobic oxidation of methane (10–30‰; Alperin *et al.*, 1988; Whiticar, 1999; Templeton *et al.*, 2006) nor as large as the very high KIEs reported for microbial oxidation of methyl halides (70‰; Miller *et al.*, 2001). However, the KIEs observed for oxidation of other C_2_ hydrocarbons [ethylene = 3‰ (Bloom *et al.*, 2000) and ethane = 8‰ (Kinnaman *et al.*, 2007)] were similar to those values reported here for C_2_H_2_ fermentation.

Consistent KIEs of 3–4‰ were achieved by San Francisco Bay sediment slurries (SFB5) during C_2_H_2_ fermentation (Fig. 1). Repeated experiments with these sediments produced similar KIEs (data not shown). These results were expected, given the observations reported above for other C_2_ metabolisms. Similar KIEs were observed during C_2_H_2_ fermentation by the pure culture SFB93 (Figs. 2 and 3). Measurements of KIEs in both growing cultures and washed cell suspensions showed identical fractionation patterns with regard to absolute values and temperature effects. This implies that incorporation of carbon into cell biomass during growth is not an influential step in controlling KIEs and further suggests that either growing or washed cell cultures may be employed to evaluate KIEs. Cultures incubated at 28°C yielded significantly higher KIEs (*p* < 0.01) than cultures incubated at 14°C; however, these differences amounted to less than 1‰. It may be that higher KIEs at warmer temperature reflect higher rates of C_2_H_2_ uptake. The preferred growth temperature for *P. acetylenicus* is 28°C (Schink, 1985), and the optimal temperature for AH activity is 50°C. (Rosner and Schink, 1995). Even so, KIEs do not always reflect differences in substrate utilization rate (Miller *et al.*, 2001), and when they do, higher rates often correlate with lower KIEs (Templeton *et al.*, 2006), in contrast to our observations.

Fermentation of C_2_H_2_ resulted in similar KIEs for Searsville Lake methanogenic enrichment SVM (Fig. 4) as those obtained for SFB5 and SFB93 (Figs. 1–3). At a fine scale, KIEs of incubations conducted at 28°C were somewhat lower for SVM (ɛ = 1.9) than for SFB5 or SFB93 incubated at 28°C (ɛ = 3.7 and 2.7, respectively). Searsville Lake enrichments were mixtures consisting of many different microorganisms including sulfate reducers when cultivated in the presence of sulfate (Miller *et al.*, 2013). In this regard they are more akin to sediment slurries than to pure cultures of C_2_H_2_ fermenters (Table 1). The composition of the microbial community in SVM was not determined; however, we hypothesize that pelobacter-like organisms containing AH may be present and could account for the similar KIE to SFB5 and SFB93.

Earlier attempts at purification of C_2_H_2_ fermenters from the milieu of Searsville Lake sediments resulted in the stable mixed culture strain SV7 (Miller *et al.*, 2013). One line of this strain (YE5) was capable of C_2_H_2_ fermentation but lacked the AH gene as determined by gene probing with degenerate primers (Miller *et al.*, 2013). This was the SV7 culture evaluated here for KIE determination (Table 1; Fig. 5). The KIE obtained from six replicate bottles (ɛ = 8.9 ± 0.8‰) was significantly (*P* < 0.01) greater than those observed for SFB5, SFB93, or SVM. This different KIE suggests that C_2_H_2_ degradation may proceed by an alternative pathway in SV7. It is possible that a reaction involving carbon occurs in the fermentation pathway of SV7 that does not occur in SVM, SFB5, or SFB93. If such a reaction discriminated in favor of ^12^C, it would contribute to the total isotope effect observed. Incubations with SV7 were conducted as growth experiments; hence, it may be that this greater fractionation reflects a different pathway of incorporation of carbon into cell biomass. However, we favor the following mechanism: Whereas both SFB93 and SV7 produced acetate as a stable end product with minor amounts of intermediate products acetaldehyde and ethanol, SV7 in addition produced formate as a stable end product (Miller *et al.*, 2013). We speculate that an unknown reaction leading to production of formate could result in additional isotopic fractionation observed in SV7. Further examination of this reaction pathway is needed to assess the veracity of this speculation. At this time, we do not know which of the six or so dominant organisms identified in SV7 via 16S rRNA sequence similarity assessment (Miller *et al.*, 2013) is conducting this putative step. Planned stable isotope probing of SV7 using ^13^C_2_H_2_ will indicate the most active participant(s) in the degradation of C_2_H_2_ (Dumont and Murrell, 2005), which may aid in delineation of this unknown reaction.

In conclusion, we successfully demonstrated that regular and persistent fractionation of stable carbon isotopes occurs during C_2_H_2_ fermentation by live sediments, sediment enrichments, and pure cultures. This is the required first step in distinguishing biological from abiotic degradation of C_2_H_2_. However, we have examined only the overall fractionation (KIE) associated with degradation of the primary substrate C_2_H_2_. In practice, synchronous measurements of δ^13^C in reactants and products for individual steps in the reaction pathway can provide additional information to the conclusions derived from measurements of δ^13^C of reactants alone. This approach has proven successful in studies of variable pathways of degradation of subsurface organic contaminants such as chlorinated ethenes (Hunkeler *et al.*, 2002; Elsner *et al.*, 2005, 2008; Hofstetter *et al.*, 2008). We propose applying this strategy to distinguish biological from abiotic C_2_H_2_ degradation in extraterrestrial environs provided each pathway results in a different KIE. For example, the Cassini mission collected material and spectroscopic data that suggest the water-rich plume emanating from Enceladus represents the composition of the aqueous environment below the moon's icy crust (Waite *et al.*, 2006; Matson *et al.*, 2007; Hansen *et al.*, 2011). If biological processes operating in the subsurface consume C_2_H_2_ and produce acetaldehyde, ethanol, and acetate, then measurement of δ^13^C of C_2_H_2_ and its degradation products in the plume should yield evidence of life below the surface of Enceladus. Measurement of δ^13^C_2_H_2_ alone would not provide unambiguous evidence of a biological process without conducting incubations of subsurface liquids containing C_2_H_2_. However, measurement of large differences between δ^13^ of C_2_H_2_ and acetaldehyde, or any other carbonaceous product (*e.g.*, ethanol, acetate, or formate) in the plume would suggest the possibility of biological fractionation. These measurements require additional analytical capabilities to those on board the Cassini spacecraft.

## Abbreviations Used

ABW: artificial mineral salts media
AH: acetylene hydratase enzyme
EA-IRMS: elemental analyzer–isotope ratio mass spectrometry
GC-C-IRMS: isotope-ratio-monitoring gas chromatography–combustion–mass spectrometry
KIEs: kinetic isotope effects
